# A Conceptual Disease Cycle Model to Link the Size of Past and Future Epidemics

**DOI:** 10.1002/ece3.71868

**Published:** 2025-07-28

**Authors:** Sam Paplauskas

**Affiliations:** ^1^ Biological and Environmental Sciences University of Stirling Stirling Scotland, UK

## Abstract

Populations of humans, animals, and plants face ongoing threats from infectious disease epidemics. While host–parasite coevolution plays a central role in shaping these dynamics, epidemics are often studied in isolation. I propose a simple “Disease Cycle” model that connects past and future epidemic sizes within the context of environmental change and evolutionary feedbacks. Drawing on recent evo‐epidemiological research, I highlight three key themes: (i) how epidemics influence the strength and direction of natural selection, (ii) how host and parasite diversity shift through evolving resistance and infectivity, and (iii) how genetic diversity in either population may affect future epidemic severity. Although gaps remain, current evidence supports this integrative model. Future research should explore how the Disease Cycle applies to non‐model organisms with low coevolutionary potential. This framework encourages a more holistic view of epidemics as dynamic outcomes of host–parasite coevolution.

## Graphical abstract

This article introduces a “Disease Cycle” model linking past and future epidemic sizes through evolutionary and environmental feedbacks. It emphasizes how epidemics drive natural selection, shape host–parasite diversity, and influence future disease severity. The framework offers a holistic perspective on epidemics as dynamic products of coevolution.

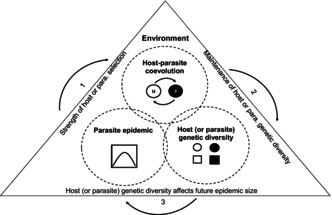

## 1 Introduction

Epidemics pose a major threat to biodiversity, global food security, and human health (Altizer et al. 2003; Jones et al. 2008; Strange and Scott 2005). In contrast to pandemics, which occur across multiple countries simultaneously, epidemics are characterized by rapidly exceeding the baseline (endemic) level of disease (Dicker 2006). For any individual parasite population, infecting hosts can have a range of individual and population‐level effects. This includes a reduction in the fitness level of individual hosts and their offspring (Lass and Ebert 2006), as well as changes in population size, genetic diversity, and community diversity (Alan Pounds et al. 2006; Altizer et al. 2003; King and Lively 2012) that can indirectly drive local extinction (Boots and Sasaki 2002). Since the negative impacts of parasite transmission in host populations can be enhanced by environmental conditions (Rogalski et al. 2017), it is essential to be able to accurately predict future epidemic size amid global change.

In contrast to wildlife disease research, human epidemiology has often revolved around the importance of acquired immunity for future epidemic size (Murphy and Weaver 2016). This is because of the key differences between vertebrate‐specific acquired and universal transgenerational innate immunity in developing a long‐lasting immunological memory (Janeway and Medzhitov 2002). Therefore, the study of environmental and ecological drivers of epidemic size, such as seasonal patterns of temperature, rainfall, and resource availability, has often been studied in wildlife disease systems (Altizer et al. 2006). However, although this research has been important for identifying risk factors, it has been unable to provide a definitive link between the size of past and future epidemics, such as how the conditions under which one outbreak occurs might affect another.

An evolutionary perspective may be useful to better understand the link between past and future epidemic size. Several studies have shown that strong host and parasite‐mediated selection, which can be attributed to a large number of infections (or epidemics), has the potential to drive rapid change in individual host resistance and parasite infectivity traits (Auld et al. 2014; Schulte et al. 2011; Thrall et al. 2012). Therefore, since larger epidemics may drive greater amounts of parasite‐mediated selection, we might expect future epidemics to be smaller as a result of host resistance.

In addition to the relationship between epidemic size and host–parasite evolutionary change, host–parasite coevolution can also affect the maintenance of host and parasite population genetic diversity (Obbard et al. 2011; Scanlan et al. 2011; Schulte et al. 2010). Whether host and parasite population genetic diversity is maintained over a long period of time or rapidly declines depends on the mode of coevolution (Brockhurst and Koskella 2013) and the underlying model of host–parasite infection genetics (Agrawal and Lively 2002). Therefore, past and future epidemics may be linked via the level of diversity in both host and parasite populations. This may be important for the relationship between past and future epidemic size because it has recently been suggested that host populations with lower genetic diversity are more likely to experience larger epidemics (Ekroth et al. 2019; Gibson and Nguyen 2020).

**FIGURE 1:**
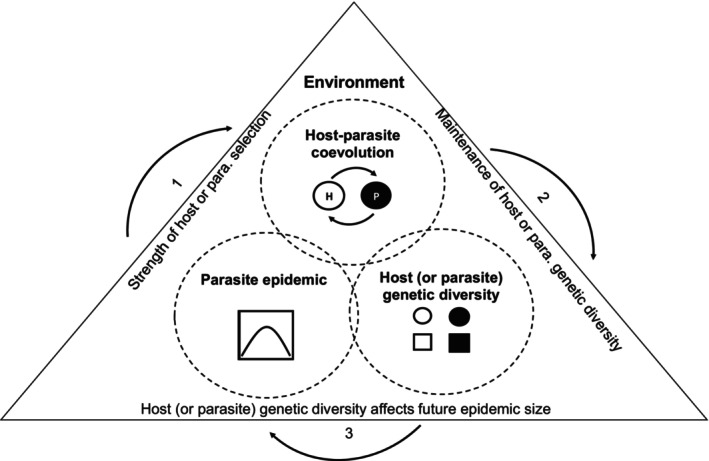
A Disease Cycle concept for linking the size of past and future epidemics. The proposed link between each component of the model (dashed circles) is shown by a numbered arrow (1–3). Specifically, I make the following predictions: (1) Epidemic size determines the strength of parasite‐mediated selection, (2) The mode of host–parasite co‐evolution determines how the level of host and parasite population genetic diversity changes over time, and (3) The level of host (or parasite) population genetic diversity determines future epidemic size. Each link in the Disease Cycle is set within the context of environmental change (triangle).

In this review, I propose a conceptual “Disease Cycle” model to link the size of past and future epidemics within a changing environment (Figure 1). I evaluate each link in the proposed model based on the current literature and consider how it would be influenced by environmental variation. Overall, I find a compelling argument for my conceptual Disease Cycle model, but there are some gaps in how the predictions from the model can be applied to non‐model organisms with a lower potential for coevolution that will need to be addressed by future research. I hope that this conceptual Disease Cycle model can encourage the scientific community to view epidemics in a coevolutionary context, in the sense of evolutionary‐epidemiology (Galvani 2003), to help develop new disease forecasting approaches.

## 2 Preconceptions and Previous Epidemiological Models

Epidemics are population‐level expressions of disease transmission. They can be defined as a rapid increase in disease prevalence (the proportion of infected individuals within a population) over time (but see Box 1). The change in the number of infected individuals per unit time can be modeled mathematically using the following equation $\frac{\mathrm{dI}}{\mathrm{dt}}=S\times \lambda -\left(\mu +\alpha \right)I$, where I is the number of infected individuals, t is time, S is the number of susceptible individuals, λ is the force of infection (the rate susceptible individuals become infected), μ is the natural mortality rate and α is the disease‐induced mortality rate. This assumes that individuals do not recover from the infected class. In this kind of model (an “Anderson and May”‐type compartmental model), the force of infection, and its underlying component parts, are crucial for shaping the size of outbreaks of infectious disease (Figure 3). However, how such Anderson and May compartmental models reflect the link between past and future epidemics remains a significant work in progress.

BOX 1 **Different ways of defining epidemic size.**

**FIGURE 2:**
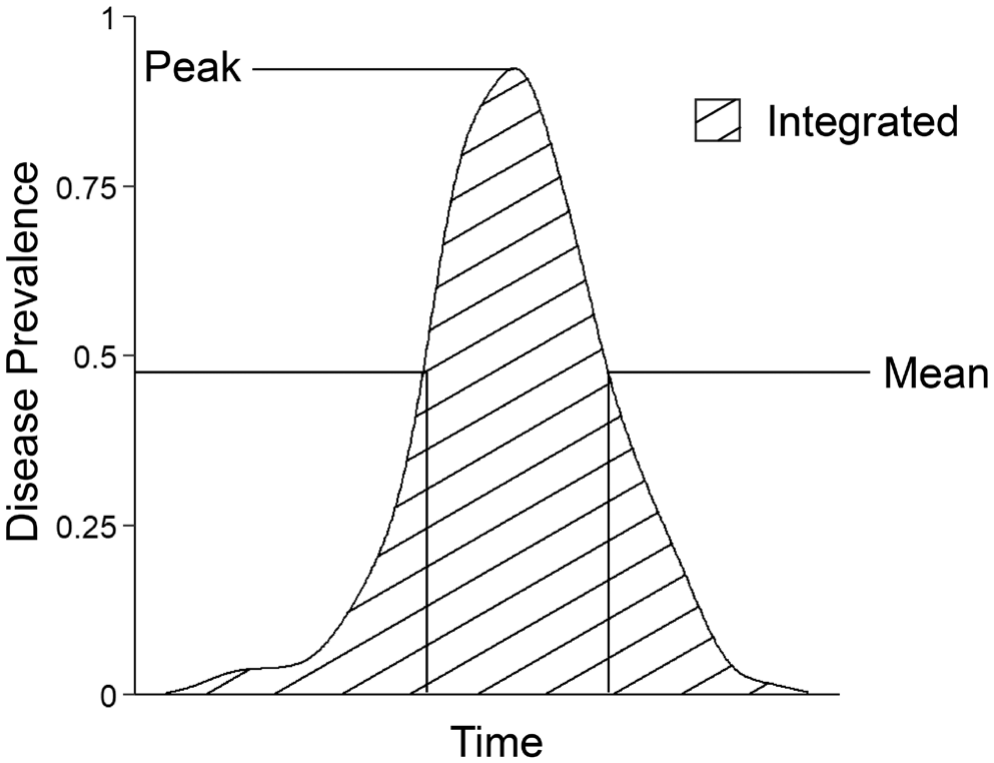
Different ways of measuring disease prevalence. This cartoon represents the change in disease prevalence over time for a hypothetical host–parasite interaction and three different ways of measuring disease prevalence. The peak disease prevalence (Peak) is the highest value for disease prevalence observed across time, and is equal to 0.9 (i.e., 90% of individuals within the population are infected). The mean disease prevalence (Mean) refers to an average disease prevalence observed between two points in time, which is indicated by the lines perpendicular to the x‐axis. The integrated disease prevalence is equal to the area underneath the curve (stripes) and represents an accumulated disease burden over time.

The most common way of defining epidemic size is as a rapid increase in the prevalence of a disease over time (Green et al. 2002). However, it can also be defined as the period in which disease prevalence surpasses a certain “disease prevalence threshold” (Duncan and Little 2007), or exceeds the endemic (i.e., baseline) level of disease prevalence (Dicker 2006). In addition, there are multiple different ways of measuring disease prevalence (Figure 2). This includes metrics such as peak prevalence, which captures a snapshot of disease prevalence at a single point in time, and other metrics such as the mean or integrated disease prevalence, which take into account some of the variation in disease prevalence over time.

Due to the inconsistency in how epidemic size is defined among studies, with many studies simply leaving epidemic size undefined (Altizer et al. 2004; Cáceres et al. 2006; Carlsson‐Granér and Thrall 2002; Duncan et al. 2006; Thrall et al. 2012), and the variety of different disease prevalence metrics (Figure 2), I propose that future studies adopt a standard measure of disease prevalence to reduce some of the challenges associated with comparing the results of different studies.

In addition, future work should try to find a standard measure for combining the size of epidemics with their impact on host populations (i.e., severity).

**FIGURE 3:**
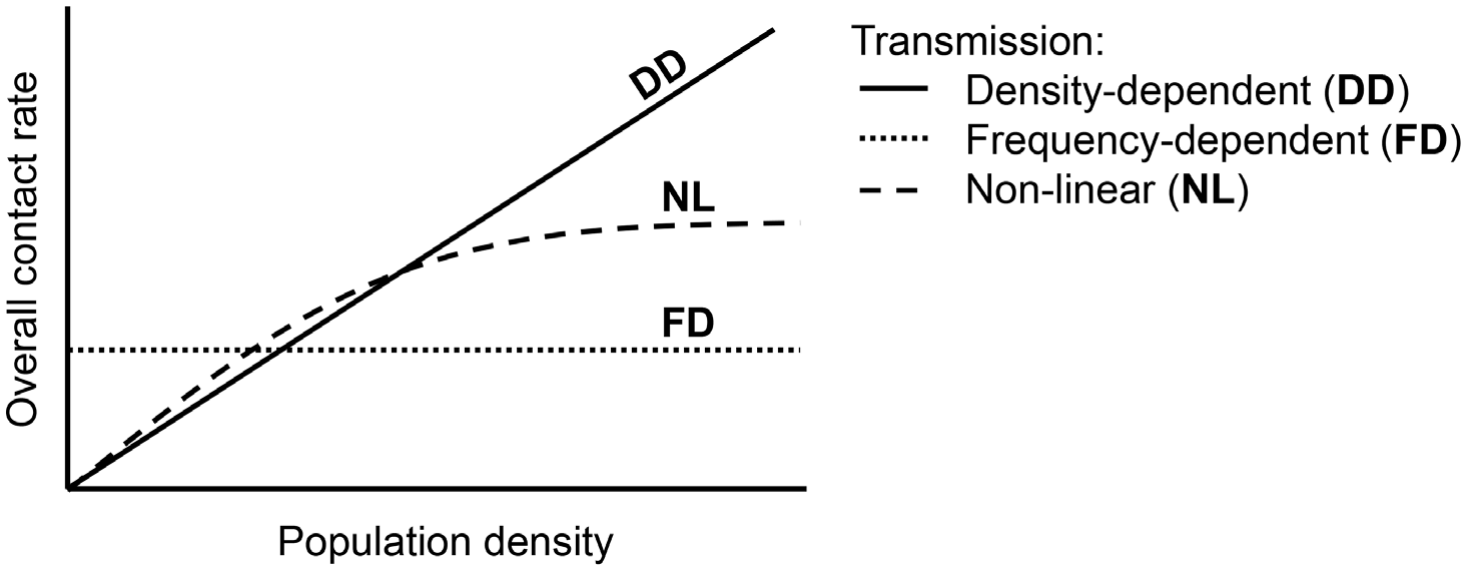
The influence that the force of infection has on the size of epidemics is transmission‐mode dependent. The force of infection for directly transmitted diseases is modeled as being density‐dependent, using the following equation: $\lambda =c\times v\times \left(I/N\right)$, where c is the host–parasite contact rate, v is the probability of transmission per contact, I is the number of infected individuals, and N is the total population size. In this case, the contact rate increases with population density (the total population size divided by the area), rather than total population size (solid black line). The host–parasite contact rate usually increases linearly with population density, but this is not always the case (dashed line). In contrast, the force of infection for both vector‐borne and sexually‐transmitted diseases is often modeled as being frequency‐dependent, using an alternative form of the same equation already shown $\lambda^{\prime }=c^{\prime }\times v\times \left(I/N\right)$. In this case, *c*′ represents an alternative version of the host–parasite contact rate, which is independent of population density. Therefore, the host–parasite contact rate is the same across different population densities (dotted line). It is worth noting that the overall contact rate refers to the average number of adequate contacts per person per unit time, where “adequate” means sufficient to transmit an infection if one of the individuals is infected. In other words, the contact rate = How many people an individual comes into contact with (in a way that can spread disease) per unit of time (Begon et al. 2006).

Currently, there are two main types of epidemiological models that account for how past and future epidemics are linked via evolution. First, statistical models that describe parasite dynamics can be incorporated as a subsidiary of a wider or more general model based on the inherent periodicity of a recurring epidemic (Axelsen et al. 2014). Second, mechanistic models that describe the evolution of the parasite population can be used to predict disease from a set of *apriori* conditions (e.g., the aforementioned Anderson and May compartmental models). Although these models have useful applications for host–parasite systems that are defined by adaptive immunity, they are less relevant for wildlife populations where there is strong antagonistic co‐evolution. In particular, this is because the evolution of resistance, from the perspectives of both the host and parasite, is seemingly always unaccounted for (Gandon et al. 2016).

## 3 Disease Arrow One: Epidemics as Engine for Host–Parasite (Co)‐Evolution

Epidemics have the potential to act as powerful engines for (co)‐evolution (Box 2). This is because epidemics can exert strong parasite‐mediated selection for the evolution of host resistance, which can drive reciprocal changes in the parasite population. Generally, we might expect that parasites are ahead in the so‐called evolutionary arms race (Schmid‐Hempel 2011), but there is considerable variation in not only the strength, but also the direction of (co)‐evolutionary change between the start and end of epidemics among studies. For example, epidemics can increase or decrease host resistance to infection (Table 1). In comparison, epidemics cannot lead to a reduction in parasite infectivity (but see Boots and Mealor 2007, for the evolution of reduced parasite infectivity during the experimental coevolution of a larval insect host—virus system).

BOX 2 **Coevolution versus (co)‐evolution.**

In host–parasite research, the term “coevolution” is often imprecisely applied to describe the evolutionary change of one or both antagonists observed during some kind of local adaptation experiment or experimental coevolution (Brockhurst and Koskella 2013). Although experimental time‐shift can be used to dissect separate host and parasite contributions to the coevolution of a shared phenotype, such as the change in disease prevalence over the course of an epidemic, the possibility that some of the evolution of one antagonist in response to the other (sensu Janzen 1980) has been captured in an attempt to purely measure evolution cannot be excluded. In other words, some part of coevolution (Janzen 1980) may have been measured along with the evolution term.

One solution to distinguish between the evolution of each antagonist and their coevolution is to measure the non‐additive component of coevolution (Paplauskas et al. 2021). This can be achieved by applying the following equation after an appropriate experiment:

$$\Delta \beta =\Delta \beta_{h}+\Delta \beta_{p}+\Delta \beta_{\mathrm{hp}}$$

This works for an experiment involving a time‐shift comparison of ancestral and future parasite transmission rates (β), but it could apply to a comparison of any infection phenotype, where:

- Δβ is the overall coevolutionary change in parasite transmission rate.
- $\Delta \beta_{h}$ is the change in parasite transmission rate owing to host evolution.
- $\Delta \beta_{p}$ is the change in parasite transmission rate owing to parasite evolution.
- $\Delta \beta_{\mathrm{hp}}$ is the change in parasite transmission rate owing to non‐additive coevolution.

The non‐additive component of host–parasite coevolution can therefore be calculated by:

$$\Delta \beta_{\mathrm{hp}}=\Delta \beta_{h}+\Delta \beta_{p}-\Delta \beta$$

By quantifying the non‐additive component of coevolution, Paplauskas et al. (2021) were able to show how variation in a mixture of environmental conditions was only able to affect the coevolutionary trajectories of replicate host–parasite ponds via their effect on the non‐additive component of coevolution through host evolution. Since the overall change observed during an experimental time‐shift can be explained by alternative amounts of host evolution, parasite evolution, or non‐additive coevolution, it might be useful to substitute coevolution for “(co)‐evolution” as a way of acknowledging the uncertainty in knowing precisely which is the major driver (Auld and Brand 2017).

**TABLE 1:** Variation in the direction of host evolutionary trajectories among different studies. For studies with differences in the direction of the evolutionary trajectories between individual host populations, the ± symbol is used. Only studies that measured host evolution in response to epidemics were included. It should be noted that this list provides insights into current biases within some of the published literature, but is by no means an exhaustive list.

Authors (year)	Host sp.	Direction of host evol.
Ameline et al. (2021)	*Daphnia magna*	+
Ameline et al. (2022)	*Daphnia magna*	+
Auld and Brand (2017)	*Daphnia magna*	±
Duffy and Sivars‐Becker (2007)	*Daphnia dentifera*	+
Duncan et al. (2006)	*Daphnia magna*	+
Ibrahim and Barrett (1991)	*Hordeum vulgare*	+
Miller and Vincent (2008)	*Oncorhynchus mykiss*	+
Mitchell et al. (2004)	*Daphnia magna*	−
Paplauskas et al. (2021)	*Daphnia magna*	±
Parker (1991)	*Amphicarpaea bracteata*	−
Strauss et al. (2017)	*Daphnia dentifera*	−
Thrall et al. (2012)	*Linum usitatissimum*	±
Zbinden et al. (2008)	*Daphnia magna*	+

The asymmetry in how the evolution of host resistance and parasite infectivity varies can possibly be explained by the relative differences in host and parasite population sizes and generation times (Schmid‐Hempel 2011), which lend themselves to rapid parasite evolution. However, this is more likely to explain differences in the magnitude of evolution, rather than its direction. Instead, variation in the direction of evolution is more likely explained by the mode of coevolution (discussed in Section 4 in more detail) and the fitness costs associated with host resistance evolution (Boots et al. 2009; Duffy and Forde 2009; Koskella 2018). One empirical study found that a resistance‐fecundity trade‐off mediated the evolution of increased host susceptibility to infection in *Daphnia* populations with a combination of large yeast epidemics and low productivity (Duffy et al. 2012). Vice versa, the opposite combination of small yeast epidemics and high productivity led to the evolution of reduced susceptibility to infection. Since the size of *Daphnia* epidemics can be determined by the level of predation risk (Duffy et al. 2019), this study confirms that the strength of antagonistic selection is relative to the strength of other (a)biotic factors.

In support of the first arrow in the Disease Cycle (Figure 1), many studies suggest that epidemic size is linked to the strength of parasite‐mediated selection (Auld and Brand 2017; Duffy et al. 2012, 2019). Until recently, empirical evidence has been limited to a single study of twenty semi‐natural outdoor mesocosms, which showed that there was a positive correlation between epidemic size and the evolution of increased host resistance to infection and parasite within‐host growth (Auld and Brand 2017). In comparison, there was no significant relationship between epidemic size and the evolution of parasite infectivity or within‐host growth. This suggests that epidemic size determines the strength of the parasite, but not host‐mediated selection. Consistent with this hypothesis, a recent meta‐analysis of 11 studies found that epidemic size does indeed determine the strength of the parasite, but not host‐mediated selection, but this finding was limited to studies of invertebrate host species (Paplauskas, unpublished). In addition, another recent study found that host resistance was futile for particularly large epidemics where the benefits of resistance were far outweighed by their fitness costs (Walsman et al. 2023).

## 4 Disease Arrow Two: How Does the Mode of Coevolution Shape Host and Parasite Genetic Diversity?

**FIGURE 4:**
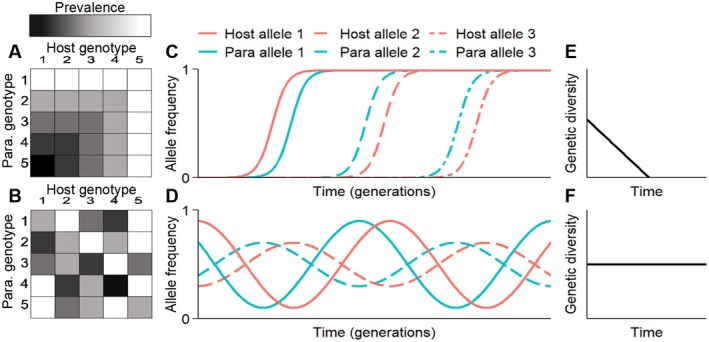
The relationship between infection genetics, the mode of coevolution, and the maintenance of genetic diversity over time. The first column shows the outcome from two cross‐infection experiments using five pairs of host and parasite genotypes that have been sampled from two hypothetical coevolution experiments. The proportion of infected hosts within each trial (prevalence) is indicated by the intensity of the color gradient. For the first cross‐infection experiment (A), a low level of genetic specificity for infection (e.g., gene‐for‐gene) means that host resistance and parasite infectivity are relative, where some host genotypes are adapted to multiple parasite genotypes and vice versa. In addition, one parasite can infect all host genotypes due to the existence of a universal virulence allele. For the second cross‐infection experiment (B), a high level of genetic specificity for infection (e.g., matching‐alleles) means that prevalence depends on pairs of host and parasite genotypes, so the color gradient is scattered across the entire grid. These two models for infection determine different coevolutionary dynamics between host and parasite alleles, as indicated by plots (C) and (D), respectively. For the first hypothetical coevolution experiment (row one), host resistance and parasite infectivity alleles are driven to fixation under arms‐race dynamics. For the second hypothetical coevolution experiment (row two), variation in the host allele frequencies lags behind those of the parasite in a negative frequency‐dependent manner (fluctuating selection dynamics). As a result, host and parasite population genetic diversity for infection decreases over time in hypothetical coevolution experiment one (row one, E), whereas host and parasite population genetic diversity for infection is maintained over time in hypothetical coevolution experiment two (row two, F). Parasite was abbreviated to Para. in panels A and B, and Para in panels C and D.

In many host–parasite systems, the nature of selection is determined by the genetic basis of infection, which in turn shapes genetic diversity in both hosts and parasites (Figure 4). Systems with low genetic specificity—such as those following the gene‐for‐gene model (Sasaki 2000; Thompson and Burdon 1992), where parasites can infect multiple host genotypes and hosts can resist multiple parasites—tend to experience directional selection (Figure 4A,C). This drives the evolution of increased host resistance and parasite infectivity through successive selective sweeps, a process known as arms race dynamics (ARD), which typically reduces genetic diversity over time (Buckling and Rainey 2002; Obbard et al. 2011; Figure 4C,E).

In contrast, high genetic specificity—as seen in systems governed by the matching‐alleles model (Bento et al. 2017; Luijckx et al. 2013), where infection success depends on precise host–parasite genotype matches—leads to negative frequency‐dependent selection (Figure 4B,D). Here, parasites are more successful against common host genotypes, which causes the frequencies of parasite genotypes to track those of their hosts. This dynamic, often referred to as fluctuating selection dynamics (FSD; Koskella and Lively 2009; Levin 1988) or Red Queen dynamics (RQD; Decaestecker et al. 2007; van Valen 1973), helps maintain genetic diversity in both populations (Figure 4D,F).

The tempo of coevolution also varies with the mode of selection. ARD typically slows the rate of coevolution as directional selection erodes genetic variation (Anderson et al. 2017; Elena et al. 1996). However, many examples of ARD come from bacteria–phage systems, where high mutation rates and short generation times naturally drive rapid evolutionary change (Brockhurst et al. 2003, 2007; Buckling and Rainey 2002; Paterson et al. 2010). By contrast, the Red Queen hypothesis predicts an accelerated evolutionary rate, driven by continual reciprocal adaptation between hosts and parasites. Empirical evidence from snail–trematode and Daphnia–parasite systems supports this idea, revealing rapid coevolution (Decaestecker et al. 2007; Koskella and Lively 2009) Still, further comparisons between coevolving populations and those evolved in isolation are needed to confirm these patterns (Paterson et al. 2010).

### 4.1 Disease Arrow Two: A Coevolutionary Continuum

ARD and RQD represent two ends of a coevolutionary continuum (Agrawal and Lively 2002; Engelstädter and Bonhoeffer 2009), with real‐world host–parasite systems falling at various points along this spectrum. In many cases, these dynamics are not mutually exclusive and may even coexist (Luijckx et al. 2013; Schmid‐Hempel and Crozier 1999; Thompson and Burdon 1992). Indeed, there is evidence for additional forms of parasite‐mediated selection, such as directional selection for increased host susceptibility, stabilizing selection (favoring intermediate resistance), and disruptive selection (favoring both highly resistant and highly susceptible host genotypes) (Duffy and Forde 2009).

Several studies demonstrate that host–parasite systems can experience multiple selection modes simultaneously (Frickel et al. 2016; Hall et al. 2011; Masri et al. 2015; Papkou et al. 2019). Furthermore, the mode of coevolution can vary not only between species, but also between populations of the same species (Betts et al. 2014) and even among replicate populations (Kortright et al. 2022). Despite these insights, our understanding of how this continuum is influenced by environmental variation remains limited.

Experimental work conducted in more realistic environmental contexts highlights how natural conditions can alter the mode of coevolution. For instance, bacteria–phage interactions follow ARD under laboratory conditions (Gómez and Buckling 2011), but shift to FSD in soil microcosms. Changes in environmental factors, such as nutrient availability and population mixing, can even reverse this shift, favoring a return to ARD (Gómez et al. 2014; Lopez Pascua et al. 2014). Similarly, mixing natural Daphnia populations disrupts FSD and leads to parasite adaptation to intermediate‐frequency host genotypes (Auld and Brand 2017).

The temporal aspect of environmental change is also important. In bacteria–phage systems, rapidly fluctuating environments hinder selective sweeps, thus constraining arms race dynamics (Harrison et al. 2013), while temperature variation can push populations in and out of evolutionary “hot” and “cold” spots (Duncan et al. 2017).

Variation in the biotic environment—including the presence of microbiota, coinfections, and parasite diversity—also plays a key role in coevolutionary outcomes. For example, in nematode systems, the presence of protective microbiota reduced the strength of FSD compared to unprotected hosts (Rafaluk‐Mohr et al. 2022). In coinfections, where hosts are infected by multiple parasites, theory predicts stronger FSD when coinfections increase fitness costs. However, outcomes can vary depending on parasite traits like fecundity and virulence (Seppälä et al. 2020).

We propose that the extent to which coinfections shift the mode of coevolution may depend on the genetic similarity among co‐infecting parasites. If similar genotypes cluster within hosts, selection remains specific, and RQD is preserved. But if parasite genotypes are dissimilar and unclustered, selection favors general host resistance, promoting ARD. More empirical research is needed to test this hypothesis.

Parasite diversity itself can alter coevolutionary trajectories. One study showed that increasing parasite diversity caused a shift from Red Queen dynamics to predominantly directional selection (Betts et al. 2018). Another found that, although bacteria evolved resistance over time when coevolving with a single phage, coevolution stalled when exposed to two divergent phage genotypes—likely due to the host's inability to evolve resistance via shared mutations (Castledine et al. 2022). This suggests that initial parasite genetic diversity can provide an early advantage to parasites that hinders long‐term coevolution.

Finally, the different stages of infection—such as host recognition, attachment, and exploitation—may be subject to different selective pressures (Agrawal and Lively 2003; Duneau et al. 2011; Fenton et al. 2012). For instance, stages that require precise host–parasite genotype matching (e.g., cell recognition, tissue localization) are likely governed by FSD. In contrast, traits like spore activation, host penetration, or resource exploitation may require less genetic specificity, and thus be governed by ARD. Additionally, preinfection by one parasite may facilitate subsequent infections by others, further reducing specificity and promoting ARD (Gopko et al. 2018).

## 5 Disease Arrow Three: Host Genetic Diversity Affects Future Epidemic Size

According to conventional wisdom, genetically homogeneous populations tend to suffer extremely large, or severe, epidemics (sensu King and Lively 2012). This is based on the assumption that there is some specificity for infection (Schmid‐Hempel and Ebert 2003) and as a result, any one parasite genotype is unlikely to transmit easily within a host population composed of multiple host genotypes. This phenomenon is well‐established as a “monoculture effect” in the agricultural literature, due to a long history of studying the devastation of crops in monoculture due to infectious diseases (Elton 1958; Garrett and Mundt 1999; Zhu et al. 2000).

Until recently, the generality of the monoculture effect in more than just crop host–parasite species associations was poorly understood. Two recent studies by Ekroth et al. (2019) and Gibson and Nguyen (2020) found that a qualitatively higher level of host population genetic diversity reduced the mean for various metrics of “parasite success” across a range of host–parasite systems. Although the definitive relationship between genetic diversity and epidemic size was not significant (Gibson and Nguyen 2020), these studies suggest that more genetically diverse host populations do indeed experience smaller epidemics, at least on average (sensu King and Lively 2012). However, the reasons why a clearly established link between host genetic diversity and epidemic size has been absent for so many years is not clearly defined.

One reason why the presence of a general monoculture effect in non‐crop hosts has taken such a long time to show is between‐study variation in the density of hosts. It has been suggested that this variation, caused by processes such as habitat fragmentation, could complicate efforts to disentangle the effects of host density from those of genetic diversity (Ekroth et al. 2019; King and Lively 2012). However, a study that measured the relative importance of these effects found that host genetic diversity had a much larger effect on disease prevalence than host density (Parsche and Lattorff 2018). In comparison, much earlier studies of the host genetic diversity–disease relationship often attributed study inconsistencies to the interactive effects of disease with other stressors, such as temperature (O'Brien and Evermann 1988). In addition, a low level of host genetic diversity does not necessarily correspond to greater susceptibility to infection. This is because host populations may have experienced different forms of selection (Box 2), leading to different levels of host resistance, depending on the genetic basis of infection (Agrawal and Lively 2002) (Box 3).

BOX 3 **What does “host genetic diversity” really mean?**

Incomparable measures of host population diversity seem to be employed across different studies. For example, a reduction in population‐level host genetic diversity as a result of inbreeding (Acevedo‐Whitehouse et al. 2003) is very different to a reduction caused by hunting (O'Brien et al. 1985; Roelke et al. 1993) or habitat fragmentation (Belasen et al. 2019). Hunting reduces genetic diversity by imposing strong directional selection for morphological (Pigeon et al. 2016) and behavioral (Leclerc et al. 2019) traits or by significantly reducing population size (Allendorf et al. 2008). In comparison, inbreeding leads to a reduction in genetic diversity mainly by increasing homozygosity (Charlesworth and Meagher 2003). Habitat loss (or fragmentation) increases the spatial separation between different sub‐populations (Cushman 2006; Leidner and Haddad 2011) and potentially may lead to reductions in gene flow and the overall genetic diversity (Aguilar et al. 2008; Frankham 2005; Honnay and Jacquemyn 2007).

### 5.1 Disease Arrow Three: Parasite Genetic Diversity Also Affects Future Epidemic Size

Although the main focus of previous research has been the difference in epidemic size between host populations with varying levels of genetic diversity (Ekroth et al. 2019; Gibson and Nguyen 2020; King and Lively 2012), parasite population diversity is also a critical factor in disease dynamics. Theory predicts that high genetic variation among parasites facilitates disease spread through epidemiological feedbacks, especially when parasite‐mediated selection is strong (Lively 2016). However, empirical work has typically focused on within‐host effects of parasite diversity (Davies et al. 2002; de Roode et al. 2005), with relatively few studies addressing population‐level outcomes. One notable exception to this is the combined study of host and parasite population genetic diversity by Ganz and Ebert (2010).

In the study by Ganz and Ebert (2010), they investigated the population‐level effect of variation in host and parasite genetic diversity on parasite prevalence in a *Daphnia* host–parasite system. They found that parasite prevalence increased in line with the number of unique parasite strains and that monocultures exposed to multiple parasite strains had both a higher mean and variability in parasite prevalence compared to polycultures (Ganz and Ebert 2010). This was important, as it suggests that higher parasite diversity increases the likelihood of encountering susceptible hosts, thereby promoting epidemic spread. Consistent with this conclusion, some studies also suggest synergistic interactions among parasites, where one strain compromises host immunity and facilitates infection by others (Karvonen et al. 2011). However, further research across diverse systems is needed to determine how generalizable these findings are.

### 5.2 Disease Arrow Three: The Identity of Host and Parasite Genotypes Matters

Alongside the effect of parasite genetic diversity, the potential influence of the identity of host and parasite genotypes on disease spread is another incredibly important, but severely undervalued area of research. One study found that variation in the identity of host and parasite genotypes explained as much as 44% of the variation observed in the likelihood of infection (Vale and Little 2009). Considering that many studies have shown the existence of complex gene‐by‐environment (G × E) interactions in both hosts and parasites, respectively (Echaubard et al. 2014; Lazzaro et al. 2008; Meixner et al. 2014; Vale and Little 2009), there is the potential for the G × E interactions of each antagonist to interact ([G_H_ × E] × [G_P_ × E]) and potentially explain an even greater proportion of the variation observed in the likelihood of infection.

### 5.3 Disease Arrow Three: Which Genes Provide the Basis for Infection?

One of the major gaps in understanding how genetic diversity affects parasite transmission, and thus epidemic size, is which genes provide the basis for host resistance and parasite infectivity traits (Ebert 2018; Ebert and Fields 2020). Although examples of these genes are rare, some studies have begun to address this knowledge gap. For example, Papkou et al. (2019) identified genomic changes in a parasite toxin gene in response to coevolution with *Caenorhabditis elegans* . In a bacteria‐phage system, coevolution drove diversification of CRISPR immunity (Guillemet et al. 2022). Likewise, Andras et al. (2020) identified a gene associated with infectivity in *Daphnia*, providing molecular support for Red Queen dynamics. In addition to identifying which genes provide the basis for infection, there are some other key questions that need to be answered, including (i) how many genes underlie host–parasite interactions, (ii) how they are organized in the genome, and (iii) what the interactions are between them (Ebert 2018).

### 5.4 Disease Arrow Three: An Epidemic‐Diversity Concept

Although recent evidence appears to show that less genetically diverse host populations are more susceptible to larger epidemics (Ekroth et al. 2019; Gibson and Nguyen 2020), this assertion is centered around the mean of various parasite success metrics. In reality, wild host populations can experience dramatic spatio‐temporal variation in epidemic size (Ericson et al. 1999; Thrall et al. 2012; Vergara et al. 2013). Therefore, understanding not only the effect of host population genetic diversity on mean metrics of parasite success, but also the *variability* in metrics of parasite success is important.

A recent study proposed an Epidemic‐Diversity Model for how host–parasite population genetic diversity can influence both the mean and variability in parasite success metrics (Paplauskas et al. 2024). By re‐analyzing the data from two previous studies (Ekroth et al. 2019; Gibson and Nguyen 2020), they found that higher levels of host population genetic diversity only reduced metrics of mean parasite success for parasites with a narrow host range, and not for parasites with a wide host range. Not only did this challenge conventional wisdom, but they also found support for the Epidemic‐Diversity conceptual model. This showed that the effect of host population genetic diversity on the variability in parasite success depends on the specific combination of the population genetic diversity of the parasite and the breadth of its host range.

## 6 Discussion

At the beginning of this review, I proposed a conceptual Disease Cycle model (Figure 1) to link the size of past and future epidemics. I evaluated each of the three main components of my proposed Disease Cycle, which included (i) the link between epidemic size and the strength of parasite‐mediated selection, (ii) changes in host–parasite diversity as a result of the (co)evolution of host resistance or parasite infectivity, and (iii) how either host or parasite population genetic diversity could impact future epidemic size.

Regarding the first prediction from my model, I found compelling evidence that epidemic size determines the strength of parasite, but not host‐mediated selection (Auld and Brand 2017; Paplauskas et al., unpublished). However, while reviewing existing research, there were two additional findings. First, in comparison to the magnitude of selection, epidemic size is unlikely to directly affect the direction of selection, unless under very specific circumstances (e.g., “resistance is futile,” Walsman et al. 2023). Instead, the direction of host and parasite‐mediated selection can be affected by the epidemic size when determined by environmental context (Duffy et al. 2012). This also links to the second finding, which is that the strength of host or parasite‐mediated selection is relative to the strength of other (a)biotic factors. For instance, other species interactions, such as competition and predation, can enhance or supress the level of disease, and thus the strength of parasite‐mediated selection (e.g., Duffy et al., 2019).

For the second and third predictions from my model, I found strong support for certain host–parasite associations, but less for others. For instance, many studies show that both the tempo and mode of host–parasite coevolution are strongly influenced by the genetic basis for infection, but the extent to which this represents other non‐model organisms with less rapid evolvability remains undetermined. Similarly, how population genetic diversity varies over a significant amount of time is unclear. Therefore, more long‐term studies of non‐model species would be needed to determine the general relationship between the mode of host–parasite coevolution and the maintenance of genetic diversity over time (but see Thrall et al. 2012 and Dewald‐Wang et al. 2022; Disease arrow two). Similarly, the third prediction from my model was well supported by recent meta‐analytical studies (Ekroth et al. 2019; Gibson and Nguyen 2020), by showing that the level of host population genetic diversity tends to be associated with a decrease in metrics of mean parasite success. However, a more recent study suggested that this effect is limited to specialist parasites with a narrow host range (Paplauskas et al. 2024).

### 6.1 Summary

The Disease Cycle offers a coevolutionary insight to how the size of past and future epidemics may be linked, within a context of broad environmental change. This is particularly relevant for outbreaks of microparasitic disease and host systems without acquired immunity. The hope and intention for my Disease Cycle model is to provide a framework for future modeling approaches that embrace epidemic disease as a recurrent episodic process and help better inform the forecasting and management of disease control strategies.

## 7 Future Directions

Future work should focus on how the coevolutionary processes outlined in the Disease Cycle could be incorporated into pre‐existing modeling frameworks. For example, adding statistical information about the Disease Cycle into epidemiological models can help to incorporate coevolution without an understanding of the underlying processes themselves. This could include general information, such as the meta‐regression coefficients from Paplauskas et al., unpublished, for the relationship between epidemic size and three axes of (co)‐evolution—host evolution, parasite evolution, and the overall “net” coevolution (i.e., Disease arrow one), or the log response ratios for the effect of host population genetic diversity on the mean and variability in parasite success (i.e., Disease arrow three; Ekroth et al. 2019; Gibson and Nguyen 2020; Paplauskas et al. 2024). The latter would also rely on estimating the levels of genetic diversity within the target population. Alternatively, it could involve collecting additional information on system‐specific rates of how host and parasite population genetic diversity change over time (i.e., Disease arrow two) from studies such as Obbard et al. (2011), Scanlan et al. (2011), and Schulte et al. (2010).

An alternative approach to adding statistical information to epidemiological models is developing mechanistic models that incorporate the different elements of the Disease Cycle. Some studies have already developed epidemiological models that incorporate the genetic basis for infection (e.g., matching alleles) and host–parasite genetic (allelic) diversity (Agrawal and Lively 2003; Springbett et al. 2003), which have even been used to examine a hypothetical “'diversity threshold” for limiting the spread of disease (Lively 2010). However, expanding the scope of this research to simulate how the genetic basis for infection affects the maintenance of genetic diversity over time (Disease arrow two) would better establish the links between past and future epidemics.

Interestingly, one final approach to incorporating the link between past and future epidemics into epidemiological models is the use of statistical inference. For example, by designing an experiment that tracks epidemics and variation in environmental conditions in a group of replicate populations over time, it is possible to “borrow” the data from these other populations to forecast epidemic size in a population of interest (Paplauskas 2025). Reproducing this experiment in other disease systems would help to test its viability as a method of rapidly predicting the spread of relatively unknown or even novel diseases.

## Author Contributions

**Sam Paplauskas:** conceptualization (lead), formal analysis (lead), investigation (lead), methodology (lead), project administration (lead), visualization (lead), writing – original draft (lead), writing – review and editing (lead).

## Conflicts of Interest

The author declares no conflicts of interest.

## Supporting information


**Data S1:** ece371868‐sup‐0001‐Supinfo.xlsx.

## Data Availability

The code for figures and the accompanying data used to support the findings of this review are openly available in Dryad at https://doi.org/10.5061/dryad.p8cz8wb2g.
