# Correction to “A Conceptual Disease Cycle Model to Link the Size of Past and Future Epidemics”

**DOI:** 10.1002/ece3.72022

**Published:** 2025-08-25

Paplauskas, S. 2025. “A Conceptual Disease Cycle Model to Link the Size of Past and Future Epidemics.” *Ecology and Evolution* 15, no. 8: e71868. https://doi.org/10.1002/ece3.71868

Figure 4 in the published article is incorrect. The colors of the squares should be reversed to align with the color gradient. The corrected version of Figure 4 is provided below.

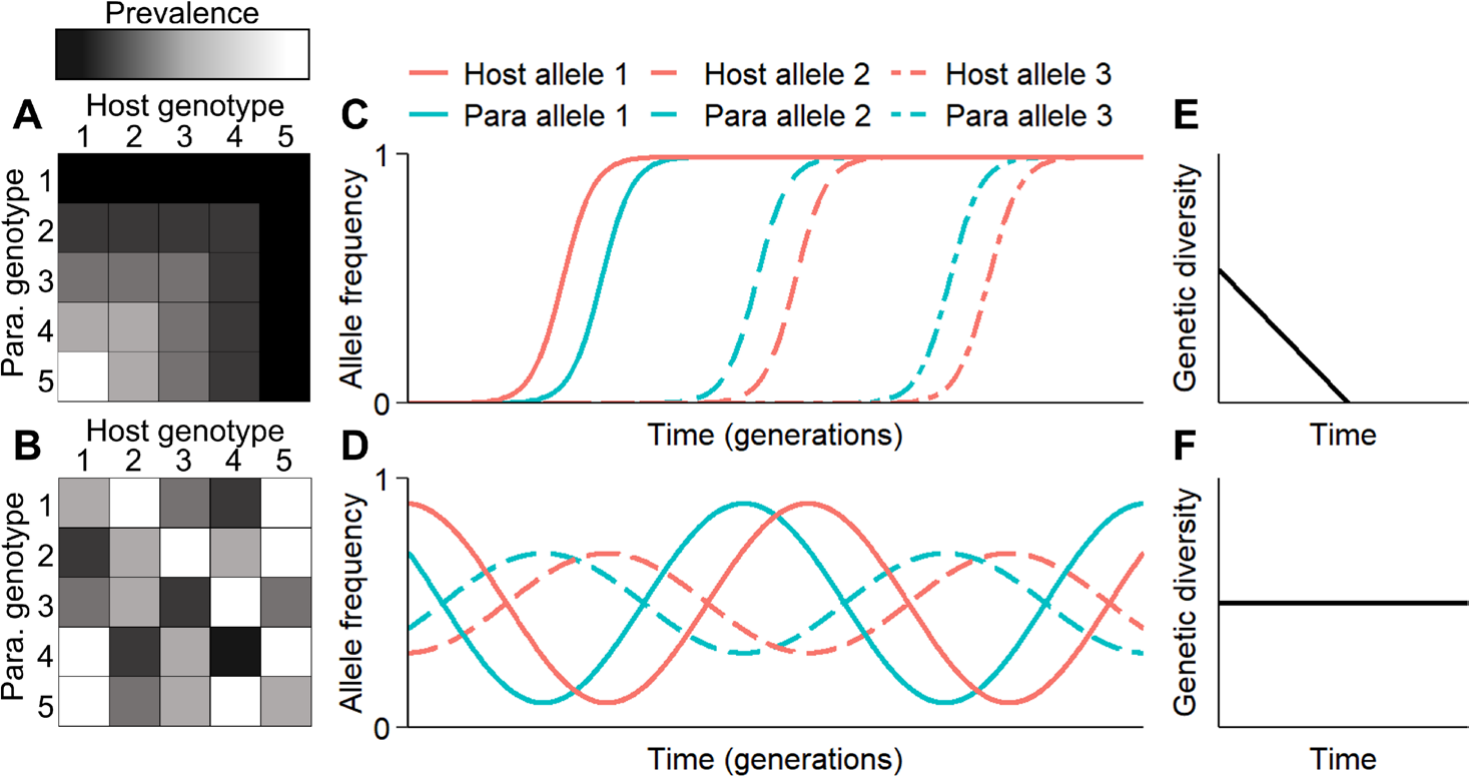

The prevalence in hosts 1 to 5 is high for parasite genotype 1, which matches the description in the figure legend “one parasite can infect all host genotypes due to the existence of a universal virulence allele.”

Additionally, the supplementary files have been updated in the published article to align with the corrected version of Figure 4.

We apologize for these errors.

